# Absence of Arsenic in the Fœtus, the Mother Having Been Poisoned by Arsenic

**Published:** 1846-11

**Authors:** 


*Absence of Arsenic in the Fœtus, the Mother having been Poisoned by Arsenic.*—In a recent case in Belgium, arsenic was detected in the fœtus carried by a female, who had been poisoned by arsenic. M. Benoist of Amiens, lately communicated to the Society of Pharmacy of that town the following case, in which the contrary was found to be the fact.

M. Benoist was charged with the examination of a young woman six months pregnant, who had poisoned herself by swallowing a considerable quantity of arsenic. The results of all his experiments perfectly demonstrated the cause of the mother's death. Not only was arsenic detected by means of Marsh's apparatus, but the poison was collected in substance on the internal surface of the stomach, and readily reduced to the metallic state.

The fœtus was at the sixth month of development, and was carefully examined in order to ascertain whether it had died in consequence of absorbing the poison which had destroyed the mother. All the experiments with Marsh's apparatus, however, gave a negative result. The combustion of the gas yielded by the apparatus was continued for upwards of an hour without obtaining a trace of arsenic.—*Ibid from Ibid.*

